# Chronic Consumption of Fructose Induces Behavioral Alterations by Increasing Orexin and Dopamine Levels in the Rat Brain

**DOI:** 10.3390/nu10111722

**Published:** 2018-11-10

**Authors:** Javier Franco-Pérez, Joaquín Manjarrez-Marmolejo, Paola Ballesteros-Zebadúa, Adriana Neri-Santos, Sergio Montes, Norma Suarez-Rivera, Miguel Hernández-Cerón, Vadim Pérez-Koldenkova

**Affiliations:** 1 Laboratorio Fisiología de la Formación Reticular, Instituto Nacional de Neurología y Neurocirugía Manuel Velasco Suárez, Ciudad de México 14269, Mexico; joaquinmanjarrez@hotmail.com (J.M.-M.); adriana.neri@ciencias.unam.mx (A.N.-S.); normanut2484@hotmail.com (N.S.-R.); 2 Laboratorio de Física Medica, Instituto Nacional de Neurología y Neurocirugía Manuel Velasco Suárez, Ciudad de México 14269, Mexico; paolabaze@gmail.com; 3 Facultad de Ciencias, Universidad Nacional Autónoma de México, Ciudad de México 04510, Mexico; 4 Departamento de Neuroquímica, Instituto Nacional de Neurología y Neurocirugía Manuel Velasco Suárez, Ciudad de México 14269, Mexico; smontes@innn.edu.mx; 5 Laboratorio de Neuropsicofarmacología, Instituto Nacional de Neurología y Neurocirugía Manuel Velasco Suárez, Ciudad de México 14269, Mexico; miguelqfbuamx@gmail.com; 6 Laboratorio Nacional de Microscopía Avanzada, Centro Médico Nacional Siglo XXI, Instituto Mexicano del Seguro Social, Ciudad de México 06720, Mexico; vadim.perez@imss.gob.mx

**Keywords:** fructose, sleep–wake cycle, locomotor activity, dopamine, orexin A, lateral hypothalamic area, ventral tegmental area

## Abstract

It has been widely described that chronic intake of fructose causes metabolic alterations which can be associated with brain function impairment. In this study, we evaluated the effects of fructose intake on the sleep–wake cycle, locomotion, and neurochemical parameters in Wistar rats. The experimental group was fed with 10% fructose in drinking water for five weeks. After treatment, metabolic indicators were quantified in blood. Electroencephalographic recordings were used to evaluate the sleep architecture and the spectral power of frequency bands. Likewise, the locomotor activity and the concentrations of orexin A and monoamines were estimated. Our results show that fructose diet significantly increased the blood levels of glucose, cholesterol, and triglycerides. Fructose modified the sleep–wake cycle of rats, increasing the waking duration and conversely decreasing the non-rapid eye movement sleep. Furthermore, these effects were accompanied by increases of the spectral power at different frequency bands. Chronic consumption of fructose caused a slight increase in the locomotor activity as well as an increase of orexin A and dopamine levels in the hypothalamus and brainstem. Specifically, immunoreactivity for orexin A was increased in the ventral tegmental area after the intake of fructose. Our study suggests that fructose induces metabolic changes and stimulates the activity of orexinergic and dopaminergic neurons, which may be responsible for alterations of the sleep–wake cycle.

## 1. Introduction

Fructose is a monosaccharide naturally present in a variety of fruits and vegetables as well as honey. However, due to its sweetness, stability, and solubility, fructose has replaced other sugars in a large number of industrialized products. Consequently, over the last several years soft drinks, fruit-flavored drinks, and sweet bakery products have become the primary food sources of fructose for the majority of the population [1]. Because of their availability, it has been observed that in the last decades the daily intake of fructose has increased, mostly in adolescent and young people [2]. 

Recent evidence has suggested that fructose consumption is a critical element in the epidemics of obesity and metabolic syndrome [3,4]. Therefore, it has been described that in humans the intake of fructose is associated with the appearance of several metabolic syndrome risk factors such as hypertriglyceridemia [5], hypertension [6,7], insulin resistance [8], and high fasting blood glucose [5,9]. Numerous studies with animal models have confirmed the metabolic findings reported in humans, and it has even been proposed that leptin resistance, inflammation, oxidative stress as well as mitochondrial damage could be underlying mechanisms in the metabolic syndrome induced by fructose [10,11,12].

Nowadays, particular emphasis has been placed on the damage that fructose can cause in the brain. Chronic feeding with fructose (4–16 weeks) was reported to induce apoptosis, reduce hippocampal neurogenesis [13], and impair hypothalamic leptin sensitivity [14], while increasing oxidative stress and neuroinflammation, and causing down-regulation of the cholinergic system in the hippocampus and cerebral cortex of rats [15]. Interestingly, recent studies have suggested that shorter periods (1–2 weeks) of fructose intake induce autophagy in rat cerebral cortex, reduce both the hippocampal weight and the expression of molecules related to cellular plasticity, and significantly increase markers of inflammation and oxidative damage in young and adult rats [16,17,18]. 

One of the effects widely described after ingesting a fructose rich-diet is the deterioration of cognitive abilities such as learning and memory. According to Ross and collaborators, rats fed with high concentrations of fructose (60%) displayed impaired spatial memory [19]. Also, the report of Yin et al. [15] showed that rats receiving a lower concentration (10% fructose solution in drinking water) exhibited a significant increase in the escape latency in the Morris water maze test. More recently, a study analyzed other cognitive parameters and showed that fructose supplementation induces an impairment of the novel object recognition memory [20].

To analyze possible underlying mechanisms in the cognitive deficits induced by fructose, we focused on the sleep–wake cycle. The sleep–wake cycle is a complex process related to a range of functions. For example, it has been described that sleep restriction has adverse effects on cognitive processes as deficits in attention, learning, and memory [21,22]. Thus, we hypothesized that cognitive impairment induced by fructose could be related to disturbances in sleep. Likewise, we decided to analyze the levels of dopamine (DA) and orexin, which are neurotransmitters involved in the regulation of the sleep–wake cycle [23]. Interestingly, it has been proposed a physiological interaction of dopaminergic and orexinergic neurons in the ventral tegmental area (VTA). The VTA is a major dopaminergic area involved in reward, feeding, arousal, and motivation [24,25,26], which receives input from the lateral hypothalamic (LH) area and can be strongly stimulated by orexins [27]. For this reason, we also analyzed the immunoreactivity of orexin A in the VTA. In our study, we describe for the first time that fructose alters the sleep–wake cycle and stimulates the activity of orexinergic and dopaminergic neurons. 

## 2. Materials and Methods

### 2.1. Animals and Treatments

Young male Wistar rats of 7–8 weeks of age weighing 180–200 g were used. Animals were maintained in individual cages in a room under controlled conditions of temperature (22 ± 2 °C) and light (12:12 light–dark cycle), with ad libitum access to food (5001 rodent diet, LabDiet, (St. Louis, MO, USA). Animals from the control group received tap water, and those from the experimental group received a 10% (*w/v*) fructose (BioBasic, Markham, ON, Canada) solution for five weeks. We selected this protocol because it has been widely described that fructose at that concentration and for similar periods of administration induces metabolic and brain alterations in animal models [13,15,28]. At the end of the treatment, the body weight gain was calculated, and some rats were perfused or euthanized by rapid decapitation. All the samples for blood analysis, chromatography, ELISA, and immunohistochemistry were obtained during the light period and between 10:00 and 12:00 h. This protocol was approved by the Institutional Committee for Care and Use of Laboratory Animals (CICUAL-INNN) and all animals were handled according to Mexican Official Norms for the production, care and use of laboratory animals (NOM-062-Z00-1999). Additionally, the Guide for the Care and Use of Laboratory Animals (NIH Guide) was revised and followed as guidelines.

### 2.2. Blood Metabolic Parameters Analysis

After rapid decapitation of the rats, 3 mL of trunk blood was collected and stored in serum blood collection tubes. The blood was centrifugated at 2000× *g* for 10 min; the serum was separated and evaluated in an automatic analyzer for clinical chemistry Cobas^®^ 6000 (Roche, Pleasanton, CA, USA). The metabolic indicators examined were glucose, triglycerides, cholesterol, alanine aminotransferase (ALT), aspartate aminotransferase (AST), alkaline phosphatase (ALP), lactate dehydrogenase (LHD), urea, and creatinine. 

### 2.3. Electrode Implantation

The rats were implanted with electrodes as previously described [29] to subsequently perform electroencephalogram (EEG) recordings and analyze the different states of the sleep–wake cycle. Briefly, one week before the end of fructose treatment the rats were deeply anesthetized via intraperitoneal administration of ketamine (Pisa, Guadalajara, JC, Mexico) (100 mg/kg) and xylazine (Pisa, Guadalajara, JC, Mexico) (10 mg/kg), and then placed in a stereotactic frame (David Kopf, Tujunga, CA, USA). Afterward, we made two small perforations in the skull to implant bipolar electrodes made of stainless-steel Teflon-coated wires (AM Systems, Sequim, WA, USA) (0.005in. diameter) with uncoated tips. An electrode implanted with a screw (Small Parts, Logansport, IN, USA) in the skull was utilized as an electrode of reference. Posterior to this, gentamicin (Pisa, Guadalajara, JC, Mexico) (40 mg/kg) and lysine clonixinate (Pisa, Guadalajara, JC, Mexico) (1 mg/kg) were intramuscularly injected as an antibiotic and analgesic, respectively.

### 2.4. EEG Recordings, Sleep Stages and Spectral Power Analysis

Subsequently, 24-h EEG recordings were carried out at the end of the fructose treatment. The EEG was obtained using the Galileo NT software (EBNeuro^®^, Firenze, Italy) and the behavior was recorded with a video camera. Later, the cortical activity and behavior were correlated to identify and analyze the different stages of the sleep–wake cycle. We estimated the total time spent in wakefulness (W; characterized by EEG desynchronization and typical behavior of an awake rat), non-rapid eye movement (NREM) sleep (characterized by slow and high voltage waves in the EEG), and rapid eye movement (REM) sleep (characterized by EEG desynchronization and the presence of rapid eye movements). The stages of the sleep–wake cycle were blindly quantified by two experimenters who did not know about the treatment [30]. For the spectral power analysis, all the EEG signals were filtered with a low-pass filter at 0.3 Hz and a high-pass filter at 70 Hz. Also, we activated the 60-Hz notch filter to discriminate the electrical noise. Every recording hour we selected episodes of W, NREM, and REM with a minimal duration of 60-s and then extracted 10-s epochs of the EEG for the following analysis. The selection of 10-s epochs allowed including EEG segments without artifacts due to cable adjustments or external noise. Using the Galileo NT software (EBNeuro^®^, Firenze, Italy), we defined the frequency bands (0.3–4 Hz for delta; 4–8 Hz for theta; 8–12 Hz for alpha; 12–30 Hz for beta; 30–70 Hz for gamma). Subsequently, the 10-s epochs of EEG were subjected to an automated analysis based on the Fast Fourier Transform (FFT) method to estimate the spectral power of each frequency band. Finally, we carried out the normalization of the data obtained by the FFT method, and results were expressed as EEG power % of control.

### 2.5. Locomotor Activity

Animals were placed in a sound-attenuated room approximately one hour before the analysis. The spontaneous locomotor activity was assessed at the end of the fructose treatment in an animal activity meter (Opto-Varimex 4, Columbus Instruments, Columbus, OH, USA). This device consists of an open-field activity chamber (45 × 45 × 20 cm) with a panel of infrared emitters (16 beams) and corresponding detectors installed purposefully along the different axes to detect the horizontal and vertical movements of the rat. Experiments were conducted placing the rats individually in the center of the chamber and allowing its free locomotion over a 10 min period. The parameters monitored were the distance traveled (cm) as well as the horizontal, ambulatory and vertical counts.

### 2.6. Enzyme-Linked Immunoassay (ELISA) Quantification of Orexin A

The rats were euthanized by rapid decapitation. The hypothalamus and brainstem (bulb, pons, and midbrain) were quickly dissected. These brain regions were analyzed because it has been described that within the hypothalamus and brainstem there are orexinergic and monoaminergic nuclei involved in the regulation of the sleep–wake cycle [23]. The hypothalamus and brainstem were homogenized using an ultrasonic processor (Sonics, Newtown, CT, USA) in lysis buffer constituted by guanidine/Tris HCl (8.2 M/82 mM) in PBS, pH 8.0, and protease inhibitor cocktail (1% *v*/*v*) (Sigma-Aldrich, St. Louis, MO, USA). The samples were centrifugated at 10,000× *g* for 10 min at 4 °C followed by the separation of the supernatants. The protein concentration in each sample was determined using the bicinchoninic acid (BCA; Sigma Aldrich, St. Louis, MO, USA) method. The quantitative detection of orexin A in brain tissue was carried out using a commercial ELISA kit (MBS264436) purchased from MyBioSource (San Diego, CA, USA). The protocol was followed exactly according to the manufacturer’s instructions. The minimum detectable concentration of orexin A was 15.6 pg/mL. The intra-assay coefficient of variation was 8% and the inter-assay 12%. After the chromogenic reaction, absorbances were measured with an ELISA microplate reader (ChroMate 4300, Awareness Technology Inc, (Palm City, FL, USA) at 450 nm within 10 min of stopping the reaction. Finally, we averaged the results of duplicate wells, and by correlating with the protein concentration, the orexin A content of each sample was calculated as pg/mg of total protein [29]. 

### 2.7. Determination of Monoamines by High-Performance Liquid Chromatography (HPLC)

The hypothalamus and brainstem samples were homogenized in a mixture of perchloric acid/sodium metabisulfite solution (1 M/0.1% *w*/*v*) followed by the centrifugation at 10,000× *g* for 10 min at 4 °C. Supernatants were separated and kept frozen at −70 °C until chromatographic analysis was performed. The contents of noradrenaline (NA), dopamine (DA), 5-hydroxyindolacetic acid (5-HIAA), and serotonin (5-HT) were analyzed by an HPLC system (LC 250, Perkin Elmer, (Waltham, MA, USA) coupled to an electrochemical detector (CC4, BAS, West Lafayette, IN, USA). We used a catecholamine analytical column (100 mm × 4.8 mm with 3-μm particle size). The mobile phase used was phosphate buffer (50 mM, pH 3.2) prepared with 0.2 mM sodium octyl sulfate, 0.1 mM EDTA and 15% (*v*/*v*) methanol. The monoamine content was calculated by extrapolating the data in a calibration curve established by standards with known concentrations [31].

### 2.8. Immunohistochemistry

To analyze the localization of orexin A in the LH and the plausible colocalization of orexin A in dopaminergic neurons of the VTA, we processed brain tissue by immunohistochemistry. A group of rats was deeply anesthetized by intraperitoneal administration of pentobarbital (100 mg/kg) (Pisa, Guadalajara, JC, Mexico) and then transcardially perfused with phosphate-buffered saline (PBS) (Sigma-Aldrich, St. Louis, MO, USA) followed by 4% paraformaldehyde (Sigma-Aldrich, St. Louis, MO, USA). Brains were removed, post-fixed in 4% paraformaldehyde for 24 h, and sequentially placed in increasing concentrations of sucrose (10, 20, 30%) for three days at 4 °C. Coronal sections (10 µm) around the LH (interaural 5.4 mm) and VTA (interaural 3.2 mm) [32] were obtained using a freezing microtome. Tissue sections were stored at −20 °C in an anti-freeze cryoprotectant solution (30% ethylene glycol, 20% glycerol in PBS, pH 7.4) (Sigma-Aldrich, St. Louis, MO, USA) until immunohistochemistry protocol was performed. Later, sections were permeabilized for 30 min with PBS containing 0.1% Triton X-100 and blocked for 60 min with 10% normal goat serum in PBS. LH slices were incubated with an antibody against orexin A (1:200, ab6214, Abcam, Cambridge, MA, USA) and VTA slices were incubated with both antibodies against orexin A and tyrosine hydroxylase (TH) as dopaminergic neuron markers (1:50, sc25269, Santa Cruz, Dallas, TX, USA) in the presence of 1% bovine serum albumin (Sigma-Aldrich, St. Louis, MO, USA) in PBS overnight at 4 °C. The next day, sections were rinsed with PBS, and LH slices were incubated with Fluorescein isothiocyanate (FITC) goat anti-rabbit IgG (1:400, ab6717, Abcam, Cambridge, MA, USA). For double immunofluorescence staining, the VTA sections were incubated with FITC goat anti-rabbit IgG (1:400, ab6717, Abcam, Cambridge, MA, USA) to detect orexin A and with DyLight 594 goat anti-mouse IgG (1:350, 35510, ThermoFisher, Waltham, MA, USA) to detect TH. Stained sections were washed with PBS, incubated with 4′,6-diamidino-2-phenylindole (DAPI) (Sigma-Aldrich, St. Louis, MO, USA) for 15 min and mounted with Gelvatol mounting medium. Images were acquired on a Nikon Ti Eclipse inverted confocal microscope equipped with an A1 imaging system; both controlled from the proprietary software NIS Elements v.4.50 (Melville, NY, USA). Imaging was performed using a 10× and 20× (dry, NA 0.5 and 0.75, respectively) objective lens. Dyes were excited in a sequential mode using the following built-in laser lines: 403 nm (DAPI), 488 nm (FITC), 561 nm (DyLight594) and a 405/488/561 dichroic mirror. Fluorochromes emissions were read in the following ranges: 425–475 nm (DAPI), 500–550 nm (FITC), 570–620 nm (DyLight594), using the filter sets provided by the manufacturer. Images were acquired and analyzed using NIS Elements v.4.50 (Melville, NY, USA) and ImageJ (Bethesda, MD, USA) Image zooming was performed by applying Nyquist sampling.

### 2.9. Statistical Analysis

All statistical analysis was performed using SigmaStat 4.0 (San Jose, CA, USA). By the values obtained from the normality and equal variance test, we calculated the *p*-value using the Mann–Whitney rank sum test or independent-samples *t*-test. Differences were considered to be significant when *p* < 0.05.

## 3. Results

### 3.1. Metabolic Parameters

As summarized in Table 1, fructose caused significant metabolic alteration increasing the concentration of glucose, triglycerides and total cholesterol in the blood. Rats fed with fructose for five weeks showed a non-significant decrease in weight gain. Likewise, the other parameters including ALT, AST, ALP, LDH, urea, and creatinine were similar in both groups. 

### 3.2. Sleep-Wake Cycle and EEG Spectral Power Analysis

**Figure 1:**
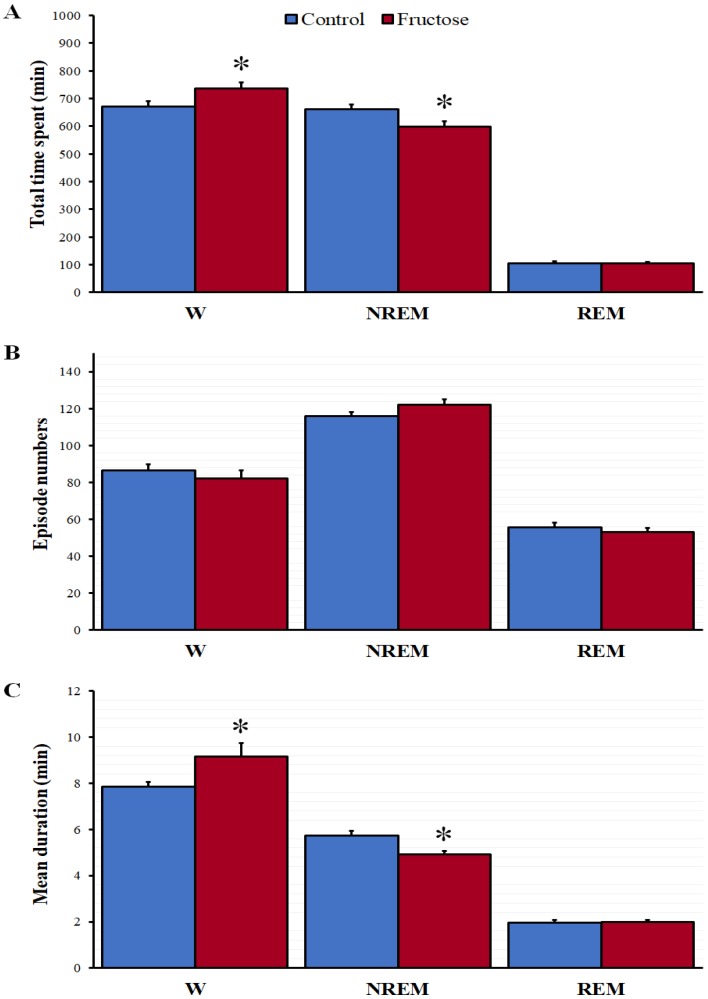
Fructose consumption modifies the sleep-wake cycle of the rat. Total time spent (**A**), number of episodes (**B**), and mean duration (**C**) of each stage following the tap water or 10% fructose ingestion for five weeks. Results are expressed as mean ± SEM, *N* = 9–10 per group. The differences between the groups were analyzed with *t*-test (* *p* < 0.05 vs. control group). W: wakefulness; NREM: non-rapid eye movement; REM: rapid eye movement.

After the chronic intake of fructose, we found a significant increase of the total time spent in W recorded over a 24 h period. By contrast, the total time spent in NREM was significantly shorter while the REM did not change (Figure 1A). The number of W, NREM, and REM episodes was similar in both groups (Figure 1B). However, the mean durations of W and NREM episodes were significantly increased and reduced, respectively (Figure 1C).

**Figure 2:**
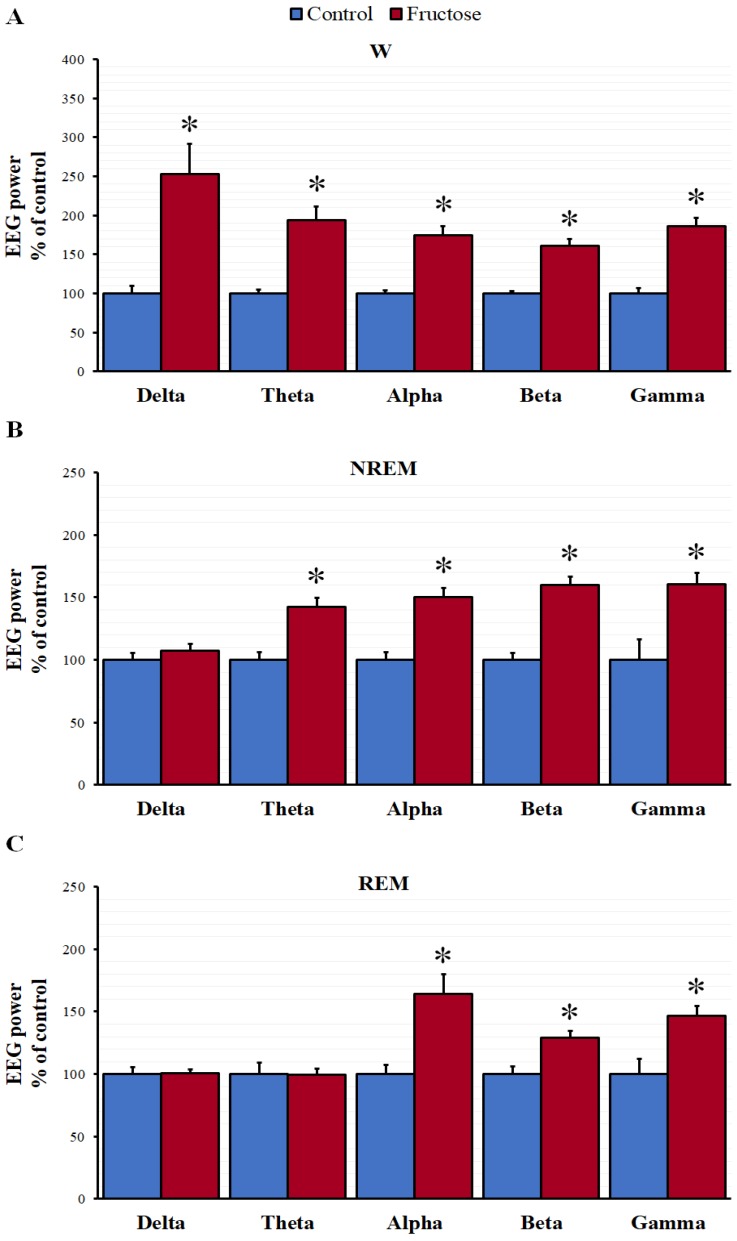
Spectral power of the electroencephalogram (EEG) in the different frequency bands (0.3–4 Hz delta; 4–8 Hz theta; 8–12 Hz alpha; 12–30 Hz beta and 30–70 Hz gamma) during W (**A**), NREM (**B**) and REM (**C**) episodes. Each bar represents the mean ± SEM of six rats per group. The results were compared statistically using a Mann–Whitney rank sum test (* *p* < 0.05 vs. control group).

Consequently, we examined the spectral power of the EEG to characterize the wake-promoting effects of fructose. As shown in Figure 2A, fructose increased the spectral power of all frequency bands during W periods. Also, fructose increased the spectral power in the theta, alpha, beta, and gamma frequency bands during the NREM (Figure 2B). In the REM sleep, we observed similar increases but only in the alpha, beta, and gamma frequencies (Figure 2C).

### 3.3. Locomotor Activity

**Figure 3:**
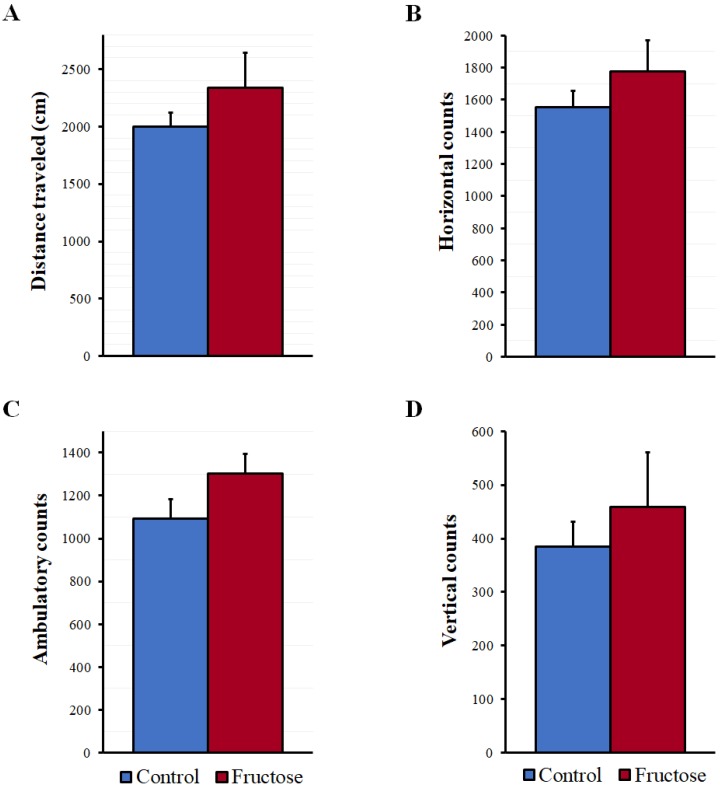
Parameters of spontaneous locomotor activity after the 10% fructose ingestion for five weeks. (**A**) Total distance traveled (cm), (**B**) horizontal activity (arbitrary units), (**C**) ambulatory activity (arbitrary units) and (**D**) vertical activity (arbitrary units) were monitored over 10 min periods. Results are expressed as mean ± SEM, *N* = 5–6 per group. The differences between the groups were analyzed with a *t*-test.

As seen in Figure 3, the distance traveled as well as the horizontal, ambulatory, and vertical counts were increased after fructose intake; however, the *t*-test indicated that the observed differences were not statistically significant.

### 3.4. Orexin A Levels

**Figure 4:**
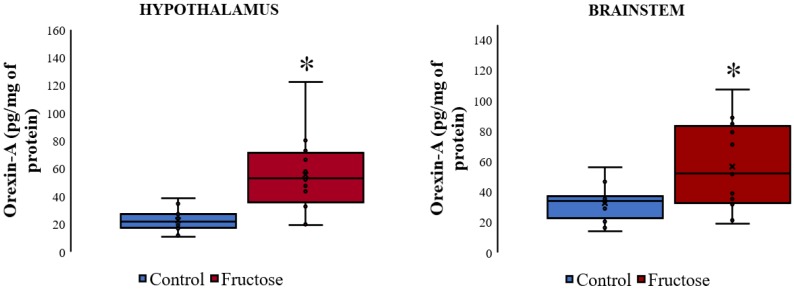
Brain levels of orexin A after feeding the rats with 10% fructose diet for five weeks. Box plots represent the median, interquartile range, and minimum and maximum values. *N* = 6 per group. The differences between the groups were analyzed with a Mann–Whitney rank sum test (* *p* < 0.05 vs. control group).

Orexin A was detectable at significant levels in the brain regions analyzed. When performing a statistical test to compare the two groups we found that chronic fructose administration induced an increase in orexin A levels in both the hypothalamus and the brainstem (Figure 4). 

### 3.5. Concentration of Monoamines

**Figure 5:**
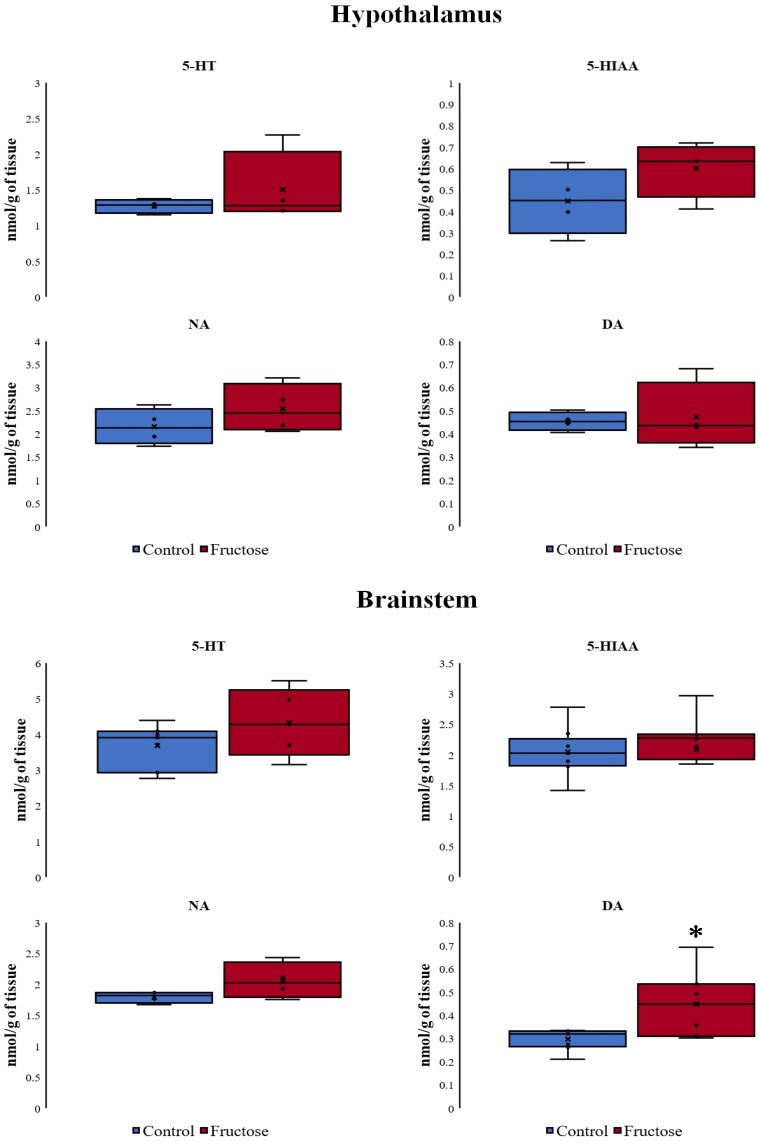
Concentration of monoamines in the hypothalamus and brainstem after the chronic ingestion of fructose. Other abbreviations used: serotonin (5-HT), 5-hydroxyindolacetic acid (5-HIAA) and noradrenaline (NA). Box plots represent the median, interquartile range, and minimum and maximum values. *N* = 4–6 per group. The differences between the groups were analyzed with a Mann–Whitney rank sum test (* *p* < 0.05 vs. control group).

We did not find significant differences when analyzed the concentration of monoamines in the hypothalamus. However, in the brainstem, all neurotransmitters and metabolites were increased, although only the increase of DA was statistically significant (Figure 5).

### 3.6. Immunohistochemical Findings

**Figure 6:**
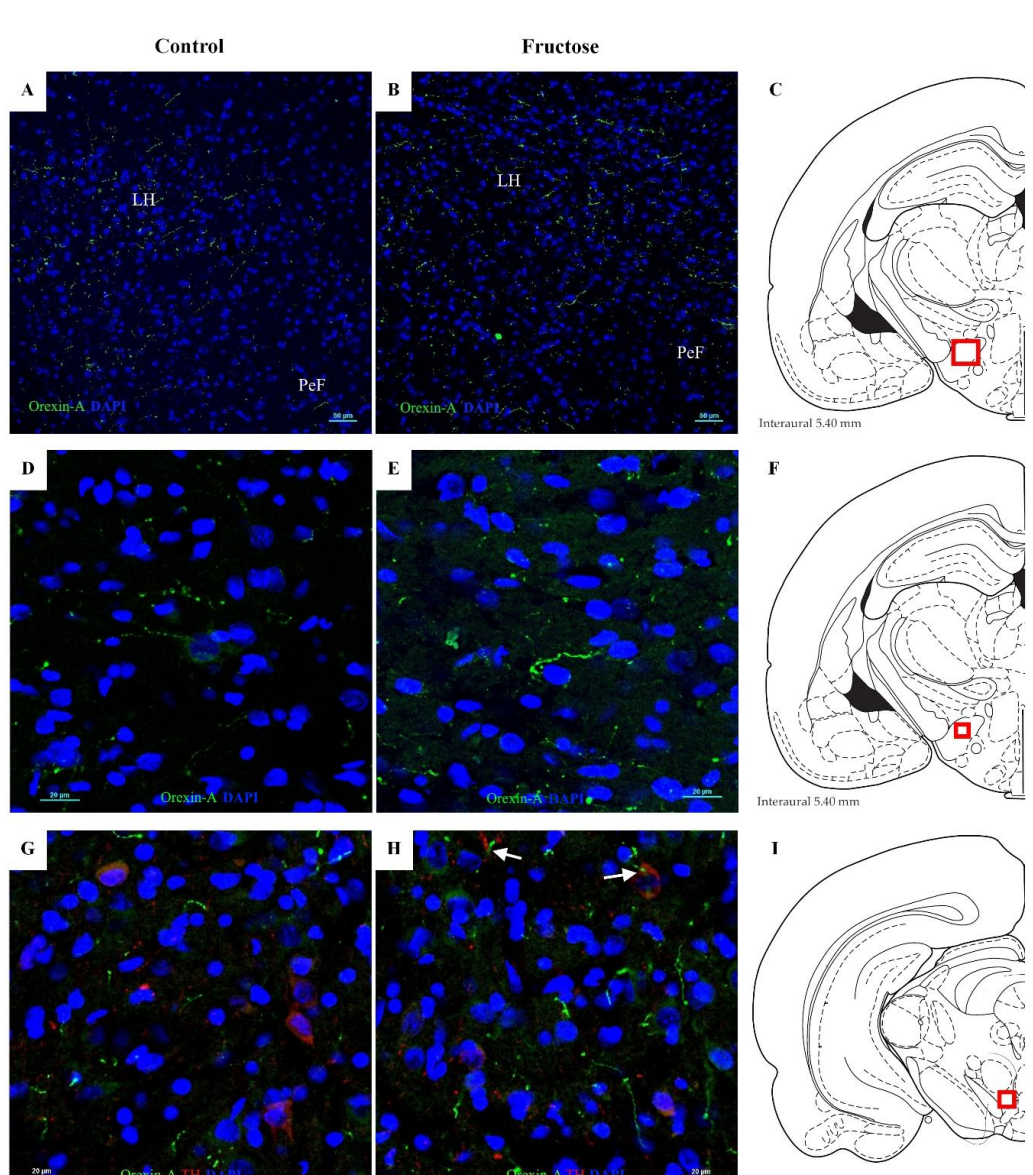
Fructose induces orexin A expression in rat brain. (**A**,**B**) Panoramic view (10×) showing the immunofluorescence staining of orexin A (green) and DAPI (blue) in the lateral hypothalamic area (LH) and perifornical nucleus (PeF). (**D**,**E**) Photomicrographs (20×) emphasizing the increase of orexin A immunoreactivity in the LH after treatment with fructose. (**G**,**H**) Ventral tegmental area (VTA) sections were immunostained simultaneously with orexin A (green), tyrosine hydroxylase (TH) (red) and DAPI (blue). Arrows indicate positive orexin A signaling that overlaps with dopaminergic neurons. Photomicrographs taken with the 20× objective were zoomed by applying Nyquist sampling (3.46×). (**C**,**F**,**I**) Diagrams showing the localization of the analyzed regions (red square) according to Paxinos and Watson [32]. *N* = 3 per group.

As expected, noticeable orexin A immunoreactivity was observed in hypothalamic regions as LH and perifornical nucleus (PeF) of control rats (Figure 6A). However, treatment with fructose for five weeks significantly increased the expression of orexin A in the mentioned regions (Figure 6B). It has been shown that orexin A causes excitation of dopaminergic neurons in the brainstem [27]. Thus, to correlate the elevated concentrations of DA and orexin A observed after the consumption of fructose, we evaluated the immunoreactivity for orexin A in the VTA. Brainstem sections were immunostained with an antibody against TH to detect dopaminergic neurons and thus to locate the VTA. In the control group, orexin A was detectable but without a clear colocalization in dopaminergic neurons (Figure 6D). On the other hand, immunoreactivity for orexin A in the VTA was increased after the chronic intake of fructose. In addition, we observed a partial overlap of orexin A signaling with dopaminergic neurons (Figure 6E).

## 4. Discussion

In the current study, we found that fructose caused disturbances in the sleep-wake cycle as well as some indicators of hyperlocomotion. Further analysis carried out showed that fructose increased the concentration of orexin A and thereby raised the levels of dopamine in the rat brain. 

Metabolic syndrome is a clustering of medical derangements that can increase the probabilities of developing numerous health problems. It has been widely described that in humans the chronic intake of fructose can lead to the appearance of typical conditions of the metabolic syndrome such as hypertriglyceridemia, hypertension, insulin resistance, and high fasting blood glucose [5,6,7,8,9]. In our study, fructose intake significantly increased the serum levels of glucose, triglycerides and total cholesterol. These findings are consistent with previous reports using animal models which have replicated the metabolic alterations found in humans [10,11,33,34]. Furthermore, a pioneering study by McNeill and Dai analyzed the concentration and duration of fructose treatment in rats and suggested that treatment with 10% fructose in drinking water for 2−6 weeks is appropriate for the establishment of metabolic syndrome risk factors [28]. Also, our data and others obtained after eight weeks of treatment have shown that weight gain is not affected by a fructose-rich diet [28,34]. However, it seems plausible that longer treatments are needed to induce significant increases in both body weight and abdominal adipose tissue weight [33].

Our results show that a fructose-rich diet modifies the sleep-wake cycle eliciting wake-promoting effects and conversely reducing the NREM sleep. These observations could be related to the cognitive function impairment associated with fructose consumption. Sleep is a highly organized process present in a wide range of animal species, and it has been proposed that sleep plays a central role in memory [35]. Numerous studies have focused on the importance of REM sleep for memory, and therefore it has been described that REM sleep deprivation alters avoidance learning, induces deficit of short-term memory, and reduces the consolidation of contextual information [36,37,38]. Moreover, it has recently been proposed that NREM sleep also play a fundamental role in the reinforcement of memory [39]. Thus, experimental studies carried out by Oyanedel and collaborators showed that total time spent in NREM positively correlated with the performance on the object–place recognition task. Also, it has been described that increased slow oscillatory activity, a hallmark of NREM, is associated with better performance in different tasks of episodic memory and novel objects recognition [40]. There are experimental models of sleep deprivation which have been very useful to understand the participation of sleep in a variety of processes including cognition and memory. However, some sleep deprivation paradigms can be aggressive and cause significant alterations. Nonetheless, there is another type of experimental manipulation called sleep restriction in which gentle manipulation is used to induce short periods of sleep deprivation but over an extended period [41]. Although we observed increases of the total time spent in W and deficits of NREM sleep after analyzing only 24-h recordings, this could be a phenomenon repeated continuously while the fructose intake continues and consequently could be considered as a paradigm of sleep restriction. Thus, one of the questions that arise is whether the alterations of the sleep-wake cycle described in this work are significant to affect the cognitive function robustly. Results obtained in rodents showed that sleep restriction is enough to cause deleterious effects on memory. It has been reported that sleep restriction for 6-h during four days selectively impaired spatial learning and decreased the number of mitotic cells in the hippocampus [42]. Interestingly, Inostroza et al. reported that short periods (80 min) of sleep restriction are enough to decline the consolidation and retrieval of spatial and temporal memories in a novel-object recognition task [43].It has been described that chronic intake of 10% fructose in drinking water induces impairment of spatial memory and novel object recognition memory [15,20]. Our results show that fructose diet significantly reduces the total time spent in NREM sleep. Therefore, we propose that sleep deficit could be one mechanism underlying the cognitive impairment induced by fructose diet.

The sleep–wake cycle is regulated by different neurotransmitters and neuronal populations located mainly in the hypothalamus and brainstem [23]. OrexinA is a neuropeptide synthesized by orexinergic neurons in the LH and PeF which has been involved in the regulation and consolidation of W episodes [44]. We found a significant increase in the orexin A levels and an evident immunoreactivity in hypothalamic regions as the LH and PeF after fructose consumption. These results are comparable to those from a previous study reporting a significant activation of orexinergic neurons in the hypothalamus of rats exposed to long-term fructose bingeing [45]. Using a retrograde tracer, Fadel and Deutch observed that orexinergic neurons from the LH densely project to midbrain regions as the VTA [46]. The rodent VTA is constituted mainly by dopaminergic and GABAergic neurons which have been involved in reward processing, feeding, arousal and motivation [24,25,26]. It has been described that dopaminergic neurons from VTA can be strongly stimulated by orexin A increasing their firing rate [27]. DA is another neurotransmitter playing a crucial role in the regulation of W. Specifically; it is well known that agonists and inhibitors of DA-transport as well as drugs promoting DA-release can also be considered as wake-promoting agents [47,48]. Our results indicate that fructose elicits an increase of orexin A and DA levels in the brainstem and visible immunoreactivity of orexin A in VTA. Given the interaction of these neurotransmitters (orexin A and DA), it is reasonable to propose that fructose first activates orexinergic neurons in the hypothalamus, and subsequently, these neurons cause stimulation of dopaminergic neurons in midbrain regions. Therefore, as a consequence of the high orexinergic and dopaminergic activity, fructose can inhibit the sleep expression and cause wake-promoting effects.

To confirm the wake enhancement caused by fructose we analyzed the EEG frequency patterns at different stages of the sleep-wake cycle. Fructose increased the spectral power of most frequency bands in W, NREM and REM periods. Interestingly, Vyazovskiy and Tobler analyzed the waking EEG and showed that during sleep deprivation there is a significant increase of the spectral power in the delta and theta frequencies and consequently proposed such alterations as markers of sleep propensity [49]. On the other hand, it seems evident that VTA influences the spectral power of different frequency bands. Experimental approaches established that bilateral lesion of the VTA provokes long-lasting suppression of theta and beta rhythms [50,51]. Other critical elements facilitating and sustaining EEG oscillatory activity are the orexins. It has been described that pharmacological lesions of nuclei with a high density of orexin receptors eliminates theta activity [52]. In addition, a recent study provided evidence explicitly describing significant decreases of theta and gamma power in an orexin knock-out model [53]. Therefore, it is tempting to speculate that high levels of orexin A found in the present study can activate VTA neurons and thus contribute, at least in part, to the increase of the EEG power observed after fructose intake. 

Finally, it is essential to analyze the possible mechanisms by which fructose consumption could be increasing the levels of orexin A in the brain. Hypothalamic neurons have a crucial function sensing external signals and regulating energy balance. Explicitly, orexinergic neurons can sense glucose and consequently trigger reversible membrane hyperpolarization inhibiting their activity. However, it has been described that fructose does not trigger any inhibitory mechanism and therefore can maintain a constant excitatory tone in the orexinergic neurons [54]. However, the more plausible mechanism involves the levels of circulating triglycerides. Chang et al. suggested that serum triglycerides modify the neuronal activity in the hypothalamus. They experimentally increased the circulating levels of lipids and later observed a substantial increase of orexins mRNA as well as increased c-Fos expression in the PeF [55]. It is noteworthy that both our present results and previous studies [10,11,33,34] have described that fructose increases the circulating levels of triglycerides. This, in turn, could be directly impacting on the activity of orexinergic neurons.

Altogether, our findings suggest that fructose induces metabolic changes and stimulates the activity of orexinergic and dopaminergic neurons, which may be a mechanism underlying the alterations of the sleep–wake cycle.

## Figures and Tables

**Table 1:** Effects of the chronic intake of fructose on metabolic parameters.

	Control	Fructose	Significance
Weight gain (g)	119.7 ± 3.3	106.2 ± 6.5	ns
Glucose (mg/dL)	111.5 ± 1.7	126.7 ± 1.5 *	*p* = 0.001
Triglycerides (mg/dL)	68.8 ± 6.8	116.4 ± 15.6 *	*p* = 0.015
Cholesterol (mg/dL)	41.5 ± 1.8	52.8 ± 3.3 *	*p* = 0.011
ALT (U/L)	83.4 ± 6.0	68.6 ± 4.8	ns
AST (U/L)	220.0 ± 17.3	197.0 ± 11.4	ns
ALP (U/L)	194.9 ± 18.2	161.1 ± 20.4	ns
LDH (U/L)	1554.9 ± 123.8	1522.6 ± 149.2	ns
Urea (mg/dL)	38.1 ± 1.5	38.3 ± 4.4	ns
Creatinine (mg/dL)	0.34 ± 0.01	0.35 ± 0.02	ns

Data are expressed as mean ± SEM, *N* = 10 per group. Data were analyzed using *t*-test (* *p* < 0.05 vs. control group). Abbreviations used: alanine aminotransferase (ALT), aspartate aminotransferase (AST), alkaline phosphatase (ALP), lactate dehydrogenase (LHD), non-significant (ns).
